# Management of Upper Lateral Cartilages (ULCs) in Rhinoplasty

**Published:** 2014-07

**Authors:** Ahmad Tavassoli Ashrafi

**Affiliations:** Faculty Member in Medical Surgical, Department of Plastic Surgery, Mazandaran, Sari

**Keywords:** Management, Upper Lateral Cartilages, Rhinoplasty

## Abstract

**BACKGROUND:**

Handling of upper lateral cartilages (ULCs) is of prime importance in rhinoplasty. This study presents the experiences among 2500 cases of rhinoplasty in the past 10 years for managing of ULCs to minimize unwilling results of the shape and functional problems of the nose.

**METHODS:**

All cases of rhinoplasties were done by the same surgeon from 2002 to 2013. Management of ULCs changed from resection to preserving the ULCs and to enhance their structural and functional roles. The techniques were spreader grafts, suturing of ULC together at the level or above the septum, using ULCs as auto-spreader flaps and very rarely trimming of ULCs unilaterally or bilaterally for making symmetric dorsal aesthetic lines. Fifty cases were operated based on this classification. Most cases were in type II and III. There were 7 cases in type I and 8 cases in type IV.

**RESULTS:**

Among most cases, the results were satisfactory although there were 8 cases for revision and among them, 2 cases had some fullness on dorsum and supra-tip because of inappropriate judgment on keeping the relationship between dorsum and tip. The problems in the shape and airways role of the nose reduced dramatically and a useful algorithm was presented.

**CONCLUSION:**

ULCs have great important roles in shape and function of nose. Preserving methods to keep these structures are of importance in surgical treatments of primary rhinoplasties. The presented algorithm helps to manage the ULCs in different anatomic types of the noses especially for surgeons who are in learning curve period.

## INTRODUCTION

Upper lateral cartilages (ULCs) are the anatomic components of the nose that not only have a major role in the anatomic shape of the nose but also are the main structure for maintaining normal passage of air through the nose and are making the lateral wall of internal valves. So the handling of ULCs is of great importance in Rhinoplasty. In older techniques of rhinoplasty, excessive resection of ULCs have made many deformities in shape (especially narrowing of dorsum) and caused many functional problems for normal passage of air through the nasal airways.^1^^-^^3^

In recent years, the trend in rhinoplasty has been changed from resection techniques to more conservative and more functional one. The survey of operated patients with airway problems revealed that the upper lateral cartilages and their preservation play a major role on preserving the normal airways functions of the nose. In the contest of these observations, from some years before different techniques for preserving the internal valve functions have developed. Among these are non-resection techniques of ULCS, splay-on graft, spreader graft, auto-spreader graft with different way of applications and combinations of them.^4^^-^^7^

The author introduce and used a new classification in his patients for managing of ULCs since 2011 depending on the amount of resection of dorsum and relative excess remaining of ULCs to desired level of dorsum. Advantages of preserving ULCs in rhinoplasties are (i) Preserving strong midvault, (ii) More flexible to use ULCs for reconstructing aesthetic dorsal lines and (iii) Preserving normal airways.

## METHODS AND MATERIALS

The author performed primary rhinoplasty on 2500 cases from 2002 to 2013. The managements of ULCs have changed and evolved through a decade of operations on the nose and the techniques changed mainly from resection to preserving the ULCs and to enhance their structural and functional roles. The main techniques were to apply spreader grafts, suturing of ULCs together at the level or above the septum, using ULCs as auto-spreader flaps and very rarely trimming of ULCs unilateral or bilaterally for making symmetric dorsal aesthetic lines.

Based on the relative excess of ULCs to desired level of dorsum, the author classified the ULCs in four groups as below (i) Type I: No cartilaginous hump, (ii) Type II : Small cartilaginous hump (1-2 mm), (iii) Type III: Moderate cartilaginous hump (3-5 mm), and (iv) Type IV: Big cartilaginous hump (>5 mm).

After resection of bony hump, the above mentioned classifications were used as a guide for individually making decision to manage the ULCs and the last 50 cases were operated on the basis of this classification. Most cases were in type II and III (15 and 20 respectively). There were 7 cases in type I and 8 cases in type IV.

In Type I cases with no cartilaginous hump, there are several options for surgically managing the UCLs which are as follow: (i) Not separating ULCs from septum, (ii) Partial separating and partial trimming, (iii) Separating of ULCs and suturing at or above septum, and (iv) Augmentation of dorsum above ULCs.

**Fig. 1:**
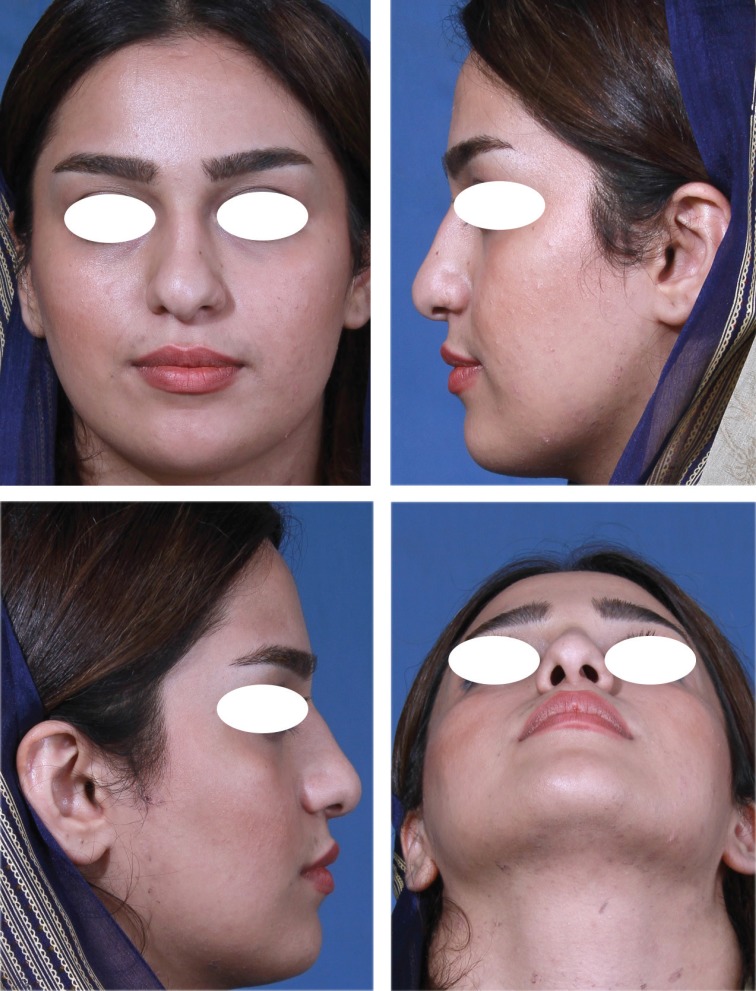
Type I. ULCs are not excess enough to be used for folding. Those could not be used for auto-spreader flap and in these situations spreader grafts were used instead

**Fig. 2:**
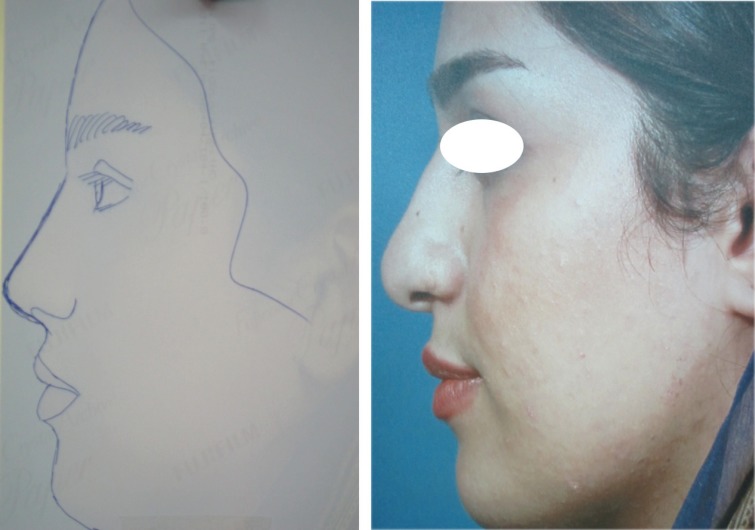
Type I: Sillouthe trimming helping to suggest how much the excess dorsum in relation to tip is and which type of ULCs has the case.

**Fig. 3:**
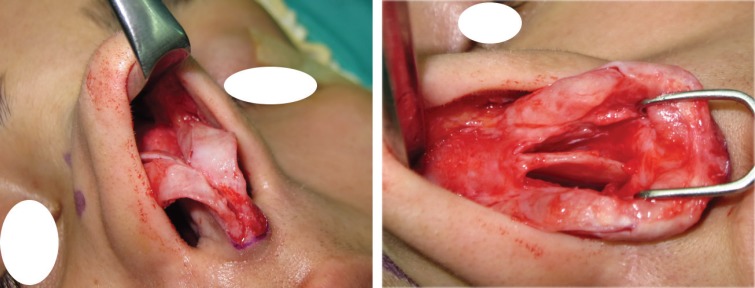
Type I: No cartilaginous hump resection without separating the ULCs from septum

**Fig. 4:**
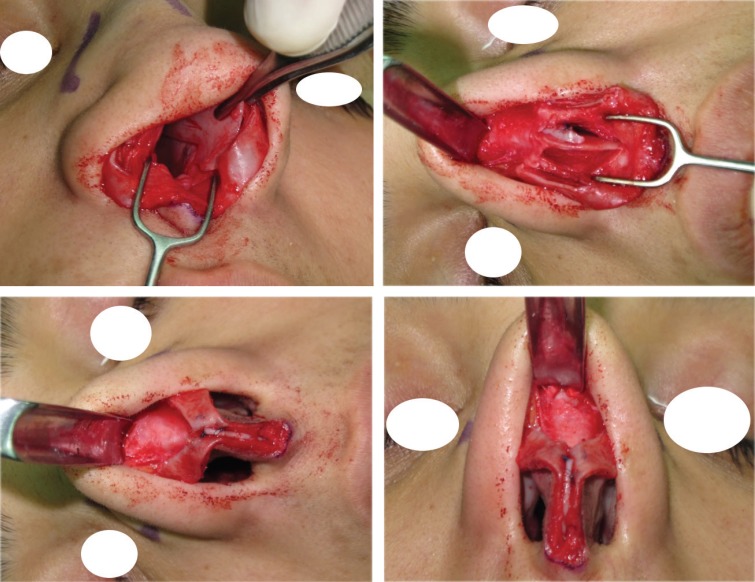
Septoplasty, tipplasty and applying unilateral spreader graft without separating the ULCs from septum

When there is no need to cut upper lateral cartilages from dorsal septum, i.e. not separating the ULCs, these are left attached and the bony hump are rasped or trimmed in relation to the height of cartilaginous hump and tip projection. In the cases with severe septal deviation or in the cases with dorsal deviation, there is a need to cut the ULCs and to close at the end of operation at or above dorsal level depending to the height differences between dorsum and tip. In some cases when the dorsum is naturally much lower than the tip, there may be a need to some kind of augmentation with dorsal graft. In type I cases as the ULCs are not excess enough to be used for folding, those could not be used for auto-spreader flap and in these situations spreader grafts were used instead (Figures 1-4). 

**Fig. 5:**
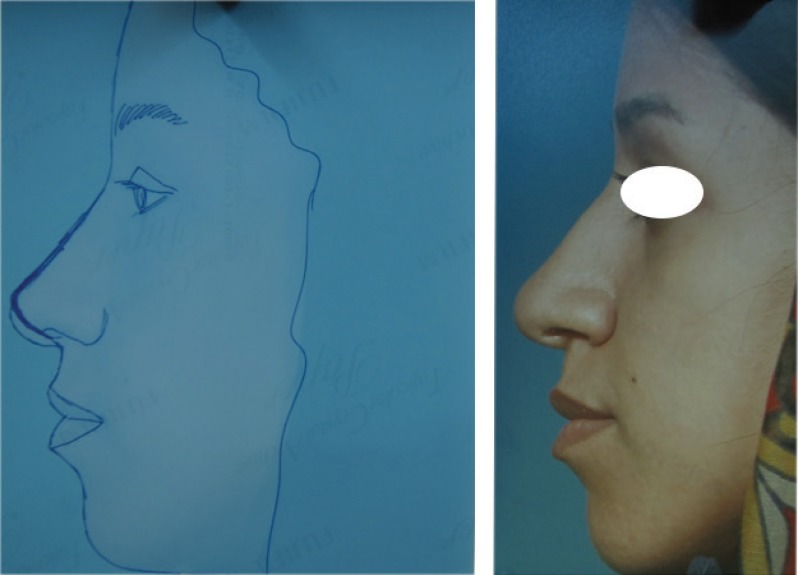
Type II: ULCs with small cartilaginous hump

**Fig. 6:**
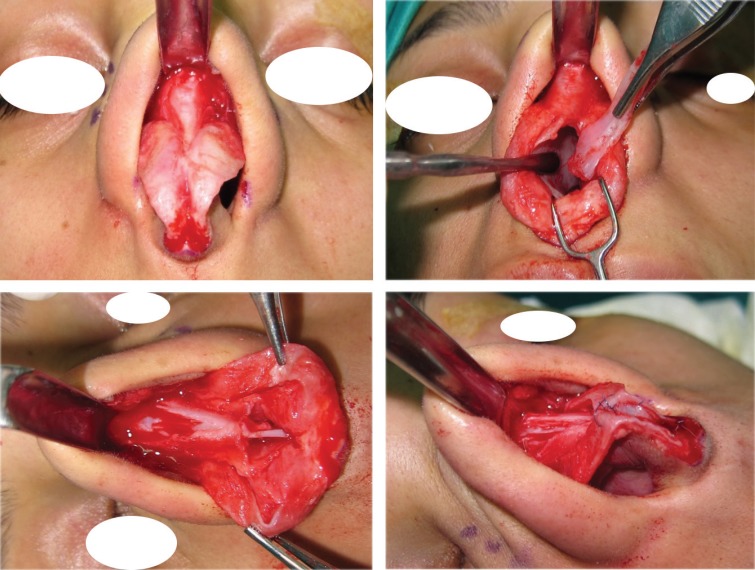
Type II: Incomplete opening of ULCs with some trimming of ULCs and septoplasty

In type II cases with small cartilaginous hump and only 1-2 mm of cartilage excess, the ULCs usually are needed to be separated from septum partially or completely but there may not be enough cartilage to be folded and used as auto-spreader flaps. In these cases, spreader grafts should be used instead or added to spreader flaps because the flaps are not strong or adequate enough. In other cases, where the ULCs are not used as flaps; these may be sutured at the level of dorsum or above dorsum for augmentation of dorsum where applicable (Figure 5 and 6).

**Fig 7:**
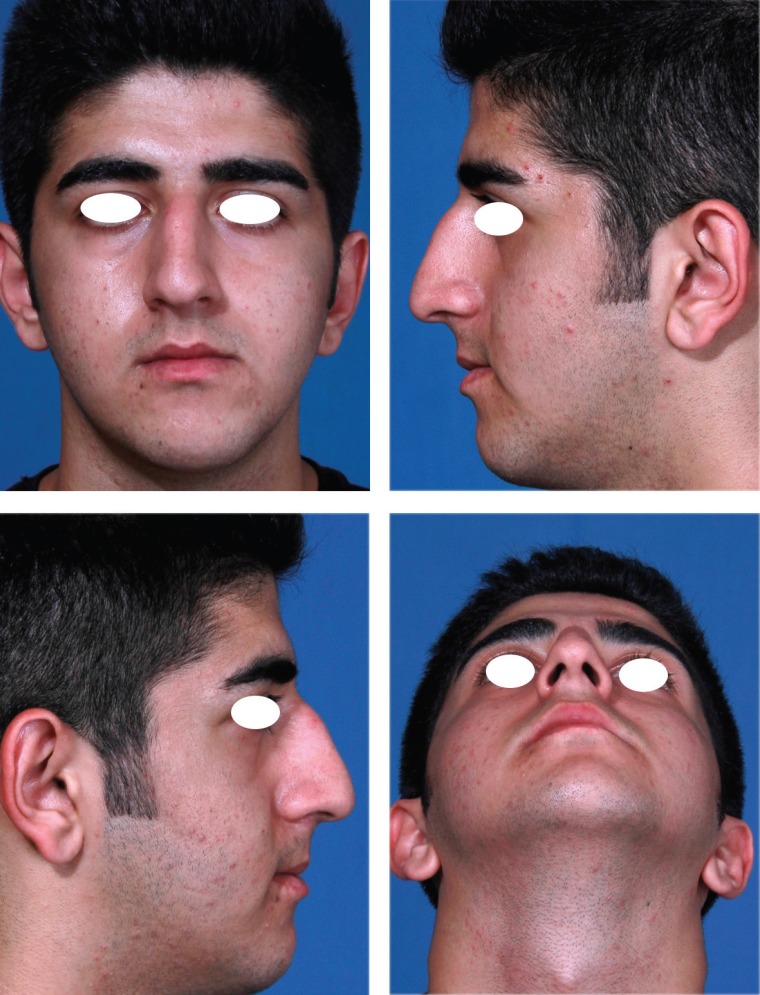
Type III: When there are excess amount of ULCs relative to the desired level of dorsum and tip, these can be folded and used as auto-spreader flaps without any need to trim the ULCs.

**Fig. 8:**
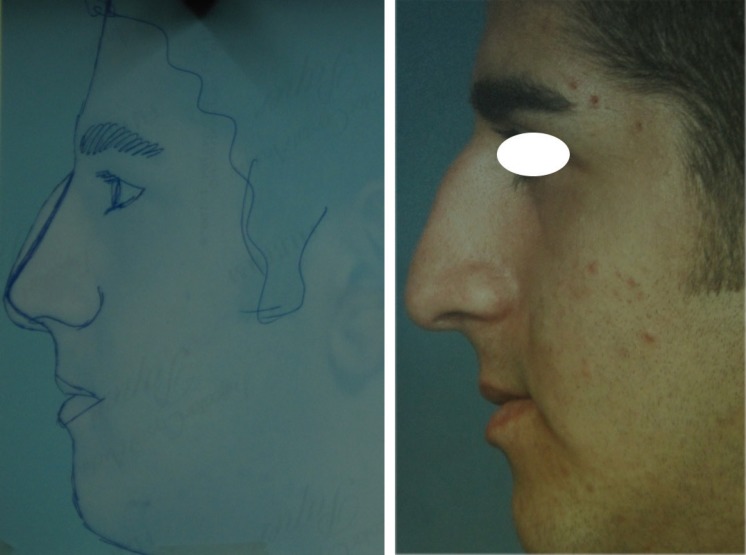
Type III: Excess ULCs between 3-5 mm

**Fig. 9:**
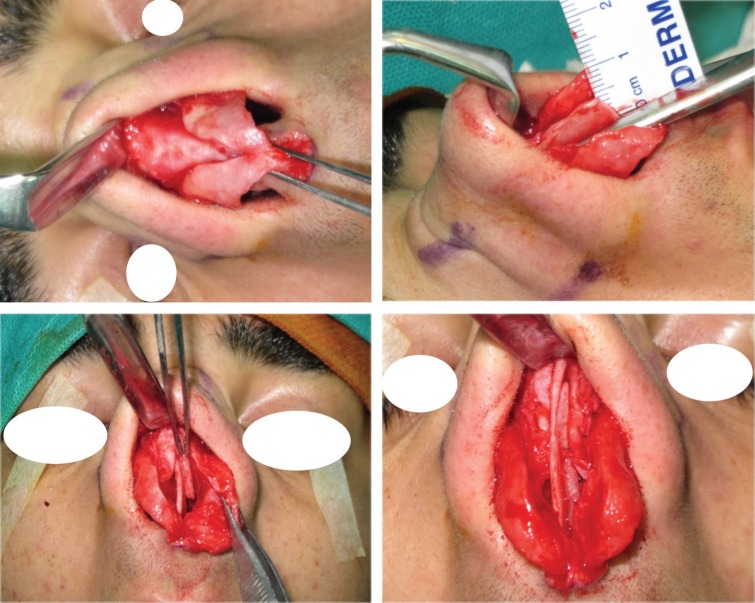
Type III: Complete opening of ULCs, left unilateral spreader graft, bilateral auto-spreader flap

In type III cases, there are moderate amount of excess cartilage between 3-5 mm and there are enough cartilage to be used as auto-spreader flaps in combination with unilateral or bilateral spreader grafts. Although, there are excess amount of ULCs relative to the desired level of dorsum and tip, however in most of the cases; these can be folded and used as auto-spreader flaps, so there is no need to trim the ULCs (Figure 7-9). In situations where more augmentation of mid-vault needed or there is severe deviation, bilateral or unilateral spreader grafts are used.

In type IV cases with excess amount of ULCs more than 5 mm, although the approach to ULCs are similar to Type III , however in some cases with wide midvault and excessive amount of ULCs, some trimming of ULCs may be needed.

## RESULTS

Fifty cases of rhinoplasty were operated on the basis of this classification since 2012 and followed up for 6-18 months. In most of the cases, the results were satisfactory, although there were 8 cases for revision. Among them, 2 cases had some fullness on dorsum and supra-tip because of inappropriate judgment on keeping the relationship between dorsum and tip.

## DISCUSSION

The trend in rhinoplasty have changed from resection techniques which were more common in closed techniques to more conservative approaches in open techniques especially in recent 2-3 decades.^1^^-^^3^

For managing the mid-vault of the nose, three important key points should be considered: First, the dorsal height in comparison to the bony part and ideal nasal tip projection, second to preserve or reconstruct the suitable dorsal width and aesthetic dorsal lines, and third to preserve internal valve function. The ULCs have been shown to have not only aesthetic roles on preserving the width and height of dorsum and making the dorsal aesthetic lines, but also have an important roles on internal valve structure and preserving the normal airways. Although, many surgeons used to trim the excess ULCs to reach to desired level of mid-vault, however the trend evolved in recent decades from resection to more conservative approaches to preserve the ULCs.^4^^-^^7^

In this study, the author classified the ULCs on the basis of the amount of the cartilaginous hump which desired to be removed. This classification would help the surgeons especially who are on the learning curve of experiences in rhinoplasty to decide easier to manage the ULCs on rhinoplasty. The author’s trend in rhinoplasty has changed from more resection techniques to more conservative approaches in management of ULCs which are reflected in the presented classifications.^2^^,^^3^^,^^5^^,^^8^^,^^9^

By using this algorithm on managements, more functional and aesthetic aspects of rhinoplasty are regarded and many procedures of routine rhinoplasties like septoplasty and spreader grafts may be applied without separating ULCs from septum especially in Type I or Type II of presented classifications. Using the excess ULCs as auto-spreader flaps not only will manage the excess ULCs, but also would improve the dorsal aesthetic lines and preserve the function of internal valves. There are some articles from many years before for conservative managements of ULCs but from recent years, the surgeons have paid more attentions on this approaches in primary rhinoplasties.^2^^,^^3^^,^^5^^,^^8^^,^^9^

In 1997 and 2007, autospreader flaps were used and was shown that this technique was simple, reproducible, and effective in shaping the dorsal midvault while preserving the function of the internal valve and should be considered when dorsal reduction is required.^10^^,^^11^

In 2004, it was demonstrated that preservation of the transverse portions of the upper lateral cartilages was essential to maintain patency of the internal nasal valve, maintain the shape of the dorsal aesthetic lines and to minimize the need for spreader grafts in primary rhinoplasty patients.^12^

In 2014, some different classifications were presented for three types of managements of upper lateral cartilages. The technique used for dorsum reconstitution was upper lateral cartilage tension spanning suture (type 1) in 65 percent, (type 2) in 25 percent, and spreader flaps (type 3) in 10 percent. A significant better dorsal aesthetic line was seen and was shown that reconstituting the nasal dorsum with repositioning of the upper lateral cartilages would provide durable cosmetic and functional results without the need for routine use of spreader grafts.^13^

The result of our study along the other studies showed that more conservative approaches to ULCs in primary rhinoplasties were a beneficial trend in rhinoplasty that improved both dorsal aesthetic lines and nasal functions. The presented classification and algorithm was useful tool for managing of the ULCs in rhinoplasty and can be used as a rough guide especially for the surgeons who are in the learning period of rhinoplasty surgery. Although the results were satisfactory in most of the cases, however more extensive studies may be needed in future for better understanding the role of ULCs and their managements in rhinoplasty.

## CONFLICT OF INTEREST

The authors declare no conflict of interest. 

## Footenote

Please cite this paper as:

Tavassoli Ashrafi A. Management of Upper Lateral Cartilages (ULCs) in Rhinoplasty. World J Plast Surg 2014;3(2):129-137.
